# Regarding the book review of Mark S. Greenburg’s ‘Handbook of neurosurgery’ (ebook), 7th edition

**DOI:** 10.1007/s00701-014-2193-7

**Published:** 2014-08-09

**Authors:** Tomasz Tykocki

**Affiliations:** Department of Neurosurgery, Institute of Psychiatry and Neurology, Sobieskiego Street, Warsaw, 02-957 Poland

Dear Editor

I read with great interest a book review by Tsitsopoulos et al. presenting their enthusiastic opinion about the value of the classic neurosurgery book, "Handbook of Neurosurgery, 7th edition," especially the electronic version. This book is undoubtedly an excellent guide for everyone interested in neurosurgery. It provides detailed information on most aspects of neurosurgery. However, after meticulous study of each chapter, some criticism might be justified. The general concept of the book is based on literature reviews related to the relevant chapter and analysed issue. This strategy requires presentation of numerous studies referring to the discussed problem, many of which contradict each other. This is very confusing for the reader, confronting many, frequently significantly different results on the same topic. The authors, whenever possible, present incontestable, strong evidence-based data, but this might refer only to a minority of the chapters. I would expect the author and excellent contributing co-authors, based on their great clinical and scientific experience, to provide more consistent, selective data with a clear final conclusion or expert opinion. A handbook referred to as a ‘vade mecum’ (Latin, “go with me”) is expected to contain guidelines rather than only mentioning a list of studies. Another confusing issue is the presentation of the epidemiology, outcomes and data-containing figures in an incoherent manner. In some chapters, information provided in one paragraph is contrary to that in other paragraphs in the same chapter. Moreover, the percentages often vary greatly (0–50 %, etc.).

I agree with Tsitsopoulos et al. that the 7th edition has been embellished with numerous high-quality color images; however, an adequate number of radiological images is still lacking. I share the opinion that the electronic version of the book facilitates its readability and accessibility, especially since it has been greatly expanded.

Despite the above-mentioned criticisms, I consider this fundamental neurosurgery book an invaluable companion for every neurosurgeon, staff members and many others involved in neurosurgery.

